# Improved NYVAC-Based Vaccine Vectors

**DOI:** 10.1371/journal.pone.0025674

**Published:** 2011-11-09

**Authors:** Karen V. Kibler, Carmen E. Gomez, Beatriz Perdiguero, Shukmei Wong, Trung Huynh, Susan Holechek, William Arndt, Victoria Jimenez, Ruben Gonzalez-Sanz, Karen Denzler, Elias K. Haddad, Ralf Wagner, Rafick P. Sékaly, James Tartaglia, Giuseppe Pantaleo, Bertram L. Jacobs, Mariano Esteban

**Affiliations:** 1 The Biodesign Institute at Arizona State University, Tempe, Arizona, United States of America; 2 Centro Nacional de Biotecnologia-Consejo Superior de Investigaciones Cientificas, Madrid, Spain; 3 Vaccine and Gene Therapy Institute of Florida, Port St. Lucie, Florida, United States of America; 4 University of Montreal, Montreal, Canada; 5 University of Regensburg, Regensburg, Germany; 6 Sanofi-Pasteur, Swiftwater, Pennsylvania, United States of America; 7 Division of Immunology and Allergy, Centre Hospitalier Universitaire Vaudois, Lausanne, Switzerland; The University of Chicago, United States of America

## Abstract

While as yet there is no vaccine against HIV/AIDS, the results of the phase III Thai trial (RV144) have been encouraging and suggest that further improvements of the prime/boost vaccine combination of a poxvirus and protein are needed. With this aim, in this investigation we have generated derivatives of the candidate vaccinia virus vaccine vector NYVAC with potentially improved functions. This has been achieved by the re-incorporation into the virus genome of two host range genes, *K1L* and *C7L*, in conjunction with the removal of the immunomodulatory viral molecule B19, an antagonist of type I interferon action. These novel virus vectors, referred to as NYVAC-C-KC and NYVAC-C-KC-ΔB19R, have acquired relevant biological characteristics, giving higher levels of antigen expression in infected cells, replication-competency in human keratinocytes and dermal fibroblasts, activation of selective host cell signal transduction pathways, and limited virus spread in tissues. Importantly, these replication-competent viruses have been demonstrated to maintain a highly attenuated phenotype.

## Introduction

Following the eradication of smallpox through the use of an efficient live vaccinia virus (VACV) for vaccination, and thereafter, through the discovery of poxviruses as recombinant vectors expressing a large range of foreign genes [1], [2], pox viruses became natural candidates for vectors to vaccinate against multiple disease-causing organisms. However, the rate of complications from wtVACV led researchers and regulators to seek pox virus vectors that would be highly attenuated to provide greater safety in humans. Three of the most well-characterized highly attenuated pox vectors have been ALVAC [3], [4], MVA [5], and NYVAC [3]. ALVAC, which is a vaccine strain of canary pox virus, is naturally attenuated in a human host, and does not replicate productively in human cells. MVA, which is also a virus that does not replicate in human cells, was passaged in chick embryo fibroblasts (CEFs) to achieve attenuation. MVA suffered several large deletions and numerous point mutations during passage in CEFs that are responsible for its attenuation. NYVAC was deleted of 18 specific open reading frames that are present in the parental Copenhagen strain, including the host range genes *K1L* and *C7L*. NYVAC is replication-incompetent in most human cells. While one of the advantages of replication-deficient viruses is their safety profiles, it has been postulated that efficacy of these viruses is limited by the failure to replicate and the attendant limitation in antigen accumulation during virus infection.

Among the many heterologous antigens that have been expressed in these replication-defective poxvirus vaccine vectors are those derived from HIV [4], [6]. Most recently, ALVAC was included in the RV144 Phase III HIV vaccine clinical trial conducted in Thailand in which a 31% efficacy rate against HIV infection was achieved after three years [7]. With the trial's encouraging demonstration that a vaccine regimen can protect to at least a limited extent, one conclusion of these findings is that the components of vaccination need to be optimized to afford protection to a large percentage of vaccinees. With this aim, in this report we describe the generation of improved NYVAC recombinants expressing the HIV antigens Env/Gag-Pol-Nef (E/GPN) from clade C. These improvements have been obtained by removal of a viral gene that inhibits type I interferon (IFN) pathways and by restoration of replication competence in human cells by inserting the *K1L* and *C7L* host range genes back into the viral genome. Our findings demonstrate that these new constructs (NYVAC-C-KC and NYVAC-C-KC-ΔB19R) have acquired new biological properties distinct from the parental NYVAC-C, which make them potentially improved vaccine vector candidates for human application.

## Materials and Methods

### Ethics Statement

All animal work at Arizona State University has been conducted in compliance with national and international guidelines. This study was approved by the Institutional Animal Care and Use Committee at Arizona State University, Tempe, Arizona, under Protocol #10-0970R.

All animal work at CNB-CSIS has been conducted in compliance with national and international guidelines and with the Royal Decree (RD 1201/2005). These studies were approved by the Ethical Committee of Animal Experimentation of Centro Nacional de Biotecnologia, Madrid, Spain, under Permit numbers 152/07 and 080030.

PBMCs for signal transduction assays were obtained from healthy blood donors recruited by the Blood Transfusion Center of Madrid; donations were approved by the ethics commission of the Center and written informed consent was obtained from the donors; all information was kept confidential by the Blood Center.

### Construction of recombinant viruses

#### Generation of NYVAC-C-ΔB19R

The plasmid transfer vector pGem-RG-B19R wm, used for the construction of the recombinant virus NYVAC-C-ΔB19R, with the *B19R* ORF deleted, was obtained by sequential cloning of five DNA fragments containing dsRed2 and rsGFP genes and *B19R* recombination flanking sequences into the plasmid pGem-7Zf(-) (Promega). The dsRed2 gene under the control of the synthetic early/late promoter was amplified by PCR from plasmid pG-dsRed2 with oligonucleotides Red2-B (5′-GAACTAGGATCCTAACTCGAGAAA-3′) (Bam HI site underlined) and Red2-N (5′-ATTAGTATGCATTTATTTATTTAGG-3′) (Nsi I site underlined) (785 bp), digested with Bam HI and Nsi I and inserted into the Bam HI/Nsi I-digested pGem-7Zf(-) to generate pGem-Red wm (3740 bp). The rsGFP gene under the control of the synthetic early/late promoter was amplified by PCR from plasmid pG-dsRed2 with oligonucleotides GFP-X (5′-CGTTGGTCTAGAGAGAAAAATTG-3′) (Xba I site underlined) and GFP-E (5′-CTATAGAATTCTCAAGCTATGC-3′) (Eco RI site underlined) (832 bp), digested with Xba I and Eco RI and inserted into plasmid pGem-Red wm previously digested with Xba I and Eco RI to obtain pGem-Red-GFP wm (4540 bp). NYVAC genome was used as the template to amplify the left flank of the *B19R* gene (364 bp) with oligonucleotides LFB19R-AatII-F (5′-TTTTTTGACGTCGAGAAAGTTAAGAAGATAC-3′) (Aat II site underlined) and LFB19R-XbaI-R (5′-TTTTTTTCTAGATCTTTATTATACGGCACTAA-3′) (Xba I site underlined). This left flank was digested with Aat II and Xba I and cloned into plasmid pGem-Red-GFP wm previously digested with the same restriction enzymes to generate pGem-RG-LFsB19R wm (4871 bp). The repeated left flank of the *B19R* gene (364 bp) was amplified by PCR from NYVAC genome with oligonucleotides LFB19R′-EcoRI-F (5′-TTTTTTGAATTCGAGAAAGTTAAGAAGATAC-3′) (Eco RI site underlined) and LFB19R′-ClaI-R (5′-TTTTTTATCGATTCTTTATTATACGGCACTAA-3′) (Cla I site underlined), digested with Eco RI and Cla I and inserted into the Eco RI/Cla I-digested pGem-RG-LFsB19R wm to generate pGem-RG-LFdB19R wm (5194 bp). The right flank of the *B19R* gene (381 bp) was amplified by PCR from the NYVAC genome with oligonucleotides RFB19R-ClaI-F (5′-TTTTTTATCGATATATACAATGCATTTTTATATAC-3′) (Cla I site underlined) and RFB19R-BamHI-R (5′-TTTTTTGGATCCAGTTCTATCATAATCATC-3′) (Bam HI site underlined), digested with Cla I and Bam HI and inserted into the Cla I/Bam HI-digested pGem-RG-LFdB19R wm. The resulting plasmid pGem-RG-B19R wm (5545 bp) was confirmed by DNA sequence analysis and directs the deletion of the *B19R* gene from the NYVAC-C genome.

The deletion mutant NYVAC-C-ΔB19R was constructed by fluorescence selection using dsRed2 and rsGFP genes as the selectable markers. 3×10^6^ BSC-40 (ATCC) cells were infected with an MOI of 0.01 of NYVAC-C and transfected 1 hour later with 6 µg DNA of plasmid pGem-RG-B19R wm using Lipofectamine (Invitrogen, San Diego, CA). 48 hours post-infection, the cells were harvested, lysed by freeze-thaw cycling, sonicated and used for recombinant virus screening. The deletion mutant was selected from progeny virus by consecutive rounds of plaque purification in BSC-40 cells during which plaques were screened for Red2/GFP fluorescence. In the first two passages viruses from selected plaques expressed both fluorescent proteins, in the next two passages viral progeny from selected plaques expressed only one fluorescent marker and in the last two passages viruses from selected plaques do not express any marker due to the loss of the fluorescent marker. The deletion mutant was detected by PCR amplifying of the *B19R* locus. The resulting NYVAC-C-ΔB19R positive virus plaques were grown in BSC-40 cells, and further passaged twice in primary CEF cells. A P2 stock was prepared in CEFs (Charles River) and used for the propagation of the virus large cultures in CEF cells, followed by virus purification by sequential centrifugation in two 36% (w/v) sucrose cushions in 10 mM Tris-HCl pH 9, and titrated by plaque assay in BSC-40 cells. The purified grown stock of virus was referred to as P3. The B8R locus of NYVAC-C-ΔB19R was deleted in a similar manner.

#### Generation of NYVAC-C-KC and NYVAC-C-KC-ΔB19R

The *C7L* and *K1L* genes were amplified from the Copenhagen genome using the following primers: NY1, 5′ GTTTGCATCGTGCTTTAACATCAATGG 3′; NY2, 5′ GTCTTACTCATTGCATCGTACGGTTGGCTTATTCCATAGTAGCTTGTG 3′; NY3, 5′ CTACTATGGAATAAGCCAACCGTACGATGCAATGAGTAAGACAATAGG 3′; and NY4, 5′ GTACCTGGCAATAGGTGATAATATGAC 3′. The “KC” fragment was then inserted into the NYVAC-C genome by *in vivo* recombination (IVR): 150 ng DNA was used to transfect MRC-5 cells (kind gift of Sanofi Pasteur) according to the manufacturer's protocol (Lipofectamine, Invitrogen), in 35 mm dishes; cells were infected with the parental virus, either NYVAC or NYVAC-C, at a multiplicity of infection (MOI) of 0.05 by adding the virus to the transfection mix. After 30 minutes of incubation, 1 ml of OptiMEM with 1% serum was added to each dish. Cells were scraped into the medium at 36 hours post-infection. Following three rounds of freeze/thaw, the IVR scrape was used to infect Vero cells (kind gift of Sanofi Pasteur) to select for viruses competent for large plaque formation in Vero cells. The same method was used to insert KC into NYVAC-C-ΔB19R.

### PCR analysis

To test for deletion of *B19R*, viral DNA was extracted by the method of SDS-Proteinase K-Phenol from BSC-40 cells mock-infected or infected at an MOI of 5 with wtNYVAC, NYVAC-C or NYVAC-C-ΔB19R. Primers LFB19R-AatII-F and LFB19R-BamHI-R spanning the *B19R* flanking regions were used for PCR analysis of *B19R* locus. The amplification reactions were carried out with Platinum Taq DNA polymerase (Invitrogen, San Diego, CA). For verification of the KC fragment, primers NY1 and NY4 were used on viral DNA obtained by standard phenol extraction from BHK-infected cells (ATCC).

### Expression of HIV-1 proteins gp120 and GPN

To verify the correct expression of the HIV-1 proteins gp120 and GPN from the viruses, monolayers of either BSC-40 or BHK cells were mock-infected or infected at an MOI of 5 with wtNYVAC, NYVAC-C, NYVAC-C-KC, NYVAC-C-ΔB19R, or NYVAC-C-KC-ΔB19R. At 48 hours post-infection, cells were lysed in Laemmli buffer, cell extracts fractionated by 10% SDS-PAGE and analyzed by Western blot using rabbit polyclonal anti-gp120 antibody (Centro Nacional de Biotecnología; diluted 1∶3000) or polyclonal anti-gag p24 serum (ARP 432, NIBSC, Centralised Facility for AIDS reagent, UK; diluted 1∶1000) followed by anti-rabbit-HRP (Sigma; diluted 1∶5000) to evaluate the expression of gp120 and GPN proteins respectively.

### Analysis of virus growth

To determine virus-growth profiles, monolayers of CEF cells grown in 12-well tissue culture plates were infected in duplicate at an MOI of 0.01 with wtNYVAC, NYVAC-C or NYVAC-C-ΔB19R. Following virus adsorption for 60 minutes at 37 C, the inoculum was removed. The infected cells were washed once with DMEM without serum and incubated with fresh DMEM containing 2% FCS at 37 C in a 5% CO_2_ atmosphere. At different times post-infection (0, 24, 48 and 72 hours), cells were removed by scraping (lysates at 5×10^5^ cells/ml), freeze-thawed three times and briefly sonicated. Virus titers in cell lysates were determined by crystal violet staining in BSC-40 cells.

To compare replication competence in human cells, NYVAC-C, NYVAC-C-KC, and NYVAC-C-KC-ΔB19R were used to infect HeLa cells (ATCC) at an MOI of 5. At 0, 6, 12, and 24 hours post-infection cells were scraped into the medium, pelleted, and the supernatant discarded. The cell pellets were resuspended in 200 µl medium and titers were obtained in BHK cells. Multi-step growth curves were done at an MOI of 0.01 in the indicated cells lines, keratinocytes (Invitrogen) and dermal fibroblasts (Lonza). IFN treatment was 1000 units/ml added for 24 hours prior to infection.

### Signal Transduction Assays

HeLa cells and human keratinocytes were infected at an MOI of 5 and allowed to incubate for 6 hours. Cell lysates were prepared by standard methods and analyzed by Western blots probed with antibodies specific to the phosphorylated forms of PKR (Cell Signaling), eIF2α (Cell Signaling), and IRF3 (Epitomic), or to the internal control, GAPDH (Abcam).

For human peripheral blood mononuclear cells (PBMCs), phospho-protein levels present in extracts of cells infected with different recombinant viruses were measured simultaneously by Cytometric Bead Array (CBA) using an LSR II flow cytometer (Becton Dickinson). In CBA technology, different bead populations with distinct fluorescence intensities had been coated with capturing antibodies specific for different analytes. These bead populations could be resolved in the fluorescence channels of the flow cytometer. After the beads had been incubated with 50 µl of sample, different analytes in the sample were captured by their corresponding beads. The protein captured beads were then mixed with phycoerythrin-conjugated detection antibodies to form sandwich complexes. Following incubation, washing and acquisition of fluorescence data, the results were generated in graphical format using the BD CBA software. The concentrations of total and phospho STAT1 were measured simultaneously using the Cell Signaling Master Buffer Kit (BD Biosciences Pharmingen, CA, USA; Cat. No. 558223). For this CBA analysis, 4×10^6^ PBMCs were mock-infected or infected at an MOI of 5 with wtNYVAC, NYVAC-C, NYVAC-C-KC, NYVAC-C-ΔB19R or NYVAC-C-KC-ΔB19R. At 4 hours post-infection, cells were removed by pipetting, centrifuged at room temperature for 5 minutes at 3000 rpm, supernatants discarded and cellular pellet resuspended in lysis buffer (Cell Signaling 10×) containing phosphatase inhibitors. After an incubation of 15 minutes at 4 C, cells were centrifuged at room temperature for 5 minutes at 13,000 rpm and supernatants transferred to new tubes. Denaturation Buffer was added and solution was mixed and denatured for 5 minutes at 100 C. Protein content in samples was measured with a bicinchoninic protein assay reagent kit (Pierce Co., Rockford, IL) and CBA analysis was performed according to manufacturer's recommendations.

### Transcriptional analysis

Ex vivo derived plasmacytoid dendritic cells (pDc, [8]) and myeloid dendritic cells (mDC, [8]) were infected with 0.1 MOI of NYVAC-C, NYVAC-C-KC, NYVAC-C-ΔB8RΔB19R and NYVAC-C-KC-ΔB8RΔB19R for 6 hours. Cells were then harvested for RNA extraction and gene array analysis. Gene array analysis was performed on BeadChips using the Illumina platform.

RNA extraction, amplification, hybridization, and scanning were performed as described previously [9]. Briefly, total RNA was purified from sorted dendritic cells using RNA extraction kits (Qiagen). RNA was quantified and assessed for quality using a spectrophotometer (NanoDrop Technologies). Total RNA was then amplified by incorporating biotin using the Illumina Total Prep RNA Amplification kit, which is based on the Eberwine amplification protocol. The biotinylated cRNA was hybridized onto Illumina Human RefSeq-8 V3 BeadChips (PBMC samples, [8]) at 58 C for 20 hours and quantified using an Illumina BeadStation 500GX scanner and Illumina BeadScan software.

Gene array analysis: scanned raw data was screened and inspected for quality. Genes with intensities below background in all samples were removed and then minimum-replaced (a surrogate-replacement policy) using the mean background value of the built-in Illumina probe controls. Normalization of chips was done by quantile-normalization.

Gene expression data was analyzed using the R software package. Genes are filtered by detection call and by variance filters to allow a reduction in the number of tests and a corresponding increase in power of the differential gene expression analysis [10]. The resulting matrix was log2 transformed and used as input for linear modeling using Bioconductor's limma package, which estimates the fold-change between predefined groups by fitting a linear model and using an empirical Bayes method to moderate standard errors of the estimated log-fold changes for expression values from each gene. P value from the resulting comparison was set to 0.05 in this analysis. Determination of commonly and uniquely regulated genes is based solely on the nominal p values and fold change.

All data is MIAME compliant and the raw data has been deposited in a MIAME compliant database.

### Measurement of apoptotic cell death by cell cycle analysis

The different stages of cell cycle and the percentage of cells with subG_0_ DNA content were analyzed by propidium iodide (PI) staining. Briefly, HeLa cells were mock-infected or infected at an MOI of 5 with NYVAC-C, NYVAC-C-KC, NYVAC-C-ΔB19R or NYVAC-C-KC-ΔB19R. At 24 hours post-infection, cells were centrifuged at room temperature for 10 minutes at 2000 rpm and supernatants discarded. Pellets were resuspended in detergent lysis buffer and cells stained with PI. After an incubation of 30 minutes at 37 C in the dark, the percentage of cells with hypodiploid DNA content was determined using an LSR II flow cytometer (Becton Dickinson). The results are expressed as fold increase in apoptotic cells with respect to uninfected cells.

### Pathogenicity in newborn mice

For studies in newborn mice, pregnant CD1 mice were purchased from Charles River at 10 days gestation. The animals were housed one animal per cage. Intracranial infections with the indicated viruses, using a total volume of 10 µL, were conducted at 48 to 72 hours post-birth of the pups (at least 10 pups per virus), using a 27-gauge needle, as previously described [11]. Animals were monitored twice daily for 14 days for morbidity and mortality.

### Mice immunization for biodistribution

BALB/c mice were purchased from Harlan. To analyze the biodistribution of virus recombinants in animals, BALB/c were immunized by intraperitoneal route (IP) with a dose of 2×10^7^ PFU/mouse of WR(TK^−^), NYVAC-C, NYVAC-C-KC, NYVAC-C-ΔB19R or NYVAC-C-KC-ΔB19R. At different times post-infection (24, 48 and 72 hours) mice were sacrificed and mouse tissues were processed for plaque assay titration. Peritoneal cells were harvested by mouse peritoneal cavity lavage with 10 ml of sterile PBS, centrifuged at room temperature for 5 minutes at 1200 rpm and stored at −70 C. Spleens, draining lymph nodes, ovaries, and livers were dissected under sterile conditions and stored at −70 C. Peritoneal cells were resuspended in 200 µl complete DMEM media and tissues from individual mice were homogenized in 200 µl complete DMEM media using an Eppendorf-fitted Dounce homogenizer. The production of infectious virus in different mouse tissues was tested by plaque assay in BSC-40 cells. The virus titer was expressed as Plaque Forming Units (PFU) per gram of protein. Protein content in tissue extracts was measured with a bicinchoninic protein assay reagent kit (Pierce Co., Rockford, IL).

Another test was also used for biodistribution by measuring levels of antigen expression in mice infected with recombinants NYVAC and NYVAC-KC expressing the luciferase marker. The recombinant viruses WR-luc and NYVAC-luc have been previously described [12], [13]. The recombinant NYVAC-KC-luc was generated by the re-insertion of KC into the genome of NYVAC-luc as described above, but using RK-13 cells (ATCC) as selection for virus-plaque isolation. Insertion of KC was confirmed by PCR and by growth characteristics in RK-13 cells.

## Results

### Generation of NYVAC-C-ΔB19R with a deletion of the virus inhibitor of type I IFN

To originally construct the parental NYVAC virus, VACV-Cop had been deleted of eighteen open reading frames (ORFs), including the TK gene [14]. For the potential use of NYVAC as an HIV vaccine vector, the genes encoding the HIV clade C antigens envelope (env), gag, polymerase (pol), and nef, were inserted into the empty TK locus to create NYVAC-C [15]. The immune response elicited by NYVAC-C was tested in mice [15], [16], monkeys [17] and humans [18], [19]. The results indicated that the vector was immunogenic, but that some improvements were needed, as the response triggered in the three different models was not strong and was largely directed against env. Moreover, priming with a DNA vector expressing the homologous HIV antigens was needed, as fewer than 40% of the human volunteers responded to two doses of the vaccine alone [18].

**Figure 1:**
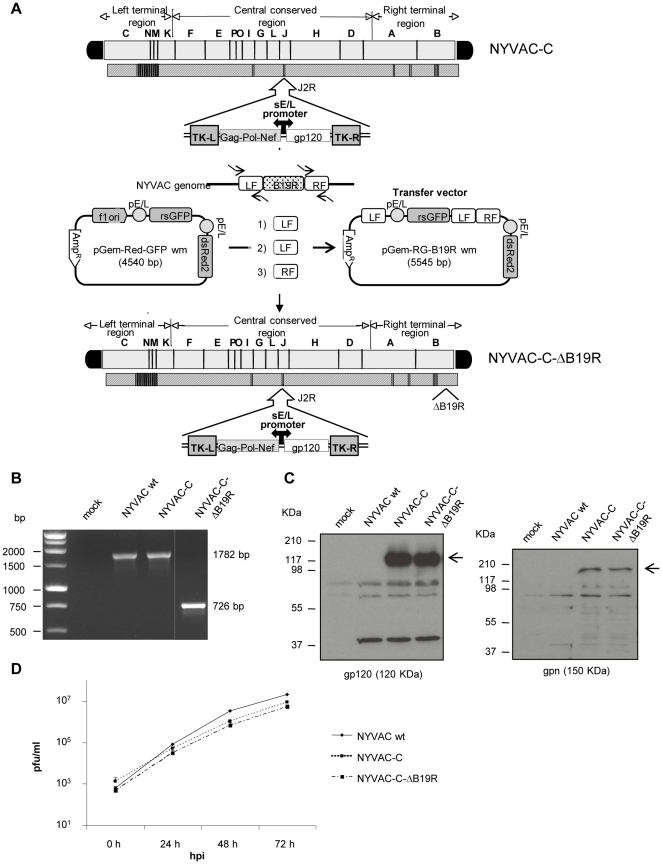
Generation and characterization of NYVAC-C-ΔB19R. **A) Scheme of construction.** The plasmid transfer vector pGem-RG-B19R wm was obtained by sequential cloning of selectable markers dsRed2 and rsGFP and *B19R* recombination flanking sequences into the plasmid pGem-7Zf. For the generation of NYVAC-C-ΔB19R, BSC-40 cells were infected at an MOI of 0.01 with NYVAC-C and transfected 1 hour later with 6 µg DNA of plasmid pGem-RG-B19R wm. 48 hours post-infection, the cells were harvested, lysed by freeze-thaw cycling, sonicated and used for recombinant virus screening. The deletion mutant was selected from progeny virus by consecutive rounds of plaque purification in BSC-40 cells during which plaques were screened for Red2/GFP fluorescence. **B) PCR analysis.** Viral DNA was extracted from BSC-40 cells mock-infected or infected at an MOI of 5 with NYVAC wt, NYVAC-C or NYVAC-C-ΔB19R. Primers LFB19R-AatII-F and LFB19R-BamHI-R spanning *B19R* flanking regions were used for PCR analysis of *B19R* locus. **C) Expression of HIV-1 antigens gp120 and GPN.** BSC-40 cells were mock-infected or infected at an MOI of 5 with NYVAC wt, NYVAC-C or NYVAC-C-ΔB19R. At 48 hours post-infection, cells were lysed in Laemmli buffer, cells extracts were fractionated by 10% SDS-PAGE and analyzed by Western blot using rabbit polyclonal anti-gp120 antibody or polyclonal anti-gag p24 serum. **D) Replication in CEF cells.** CEF cells were infected at an MOI of 0.01 with NYVAC wt, NYVAC-C or NYVAC-C-ΔB19R. At different times post-infection (hpi, 0, 24, 48 and 72 hours), cells were harvested, freeze-thawed three times and sonicated. Virus titers in cell lysates were determined by crystal violet staining in BSC-40 cells.

To improve the vector, we compiled a list of vaccinia virus genes that are present in the NYVAC genome that are known to interrupt or abrogate host immune system pathways, particularly those of the interferon system [20]. We selected for deletion in the NYVAC genome the gene *B19R* (*B18R* in WR strain), which encodes a soluble type I IFN binding protein [21], and *B8R*, which encodes a soluble type II IFN binding protein [22]. NYVAC-C-ΔB19R was constructed by replacing the *B19R* gene with a GFP/dsRed selection cassette through the use of *in vivo* recombination (IVR). The viral genome then resolves to remove the duplicate coding regions, leaving an empty locus in the place of the targeted gene. The process to insert the cassette and remove it is illustrated in Figure 1A. The *B8R*, *B19R* double deletion was prepared in a similar manner, starting with NYVAC-C-ΔB19R. Characterization of NYVAC-C-ΔB19R is shown in Figures 1 B–D. Similar data has been obtained for the double mutant (data not shown). Deletion of *B19R* was verified by PCR (Fig. 1B) and expression of the HIV antigens was verified by Western blot (Fig. 1C). The deletion mutant NYVAC-C-ΔB19R replicated in CEFs, as shown in Figure 1D, to titers similar to those of the parental NYVAC-C.

### Generation of replication-competent NYVAC Vectors

**Figure 2:**
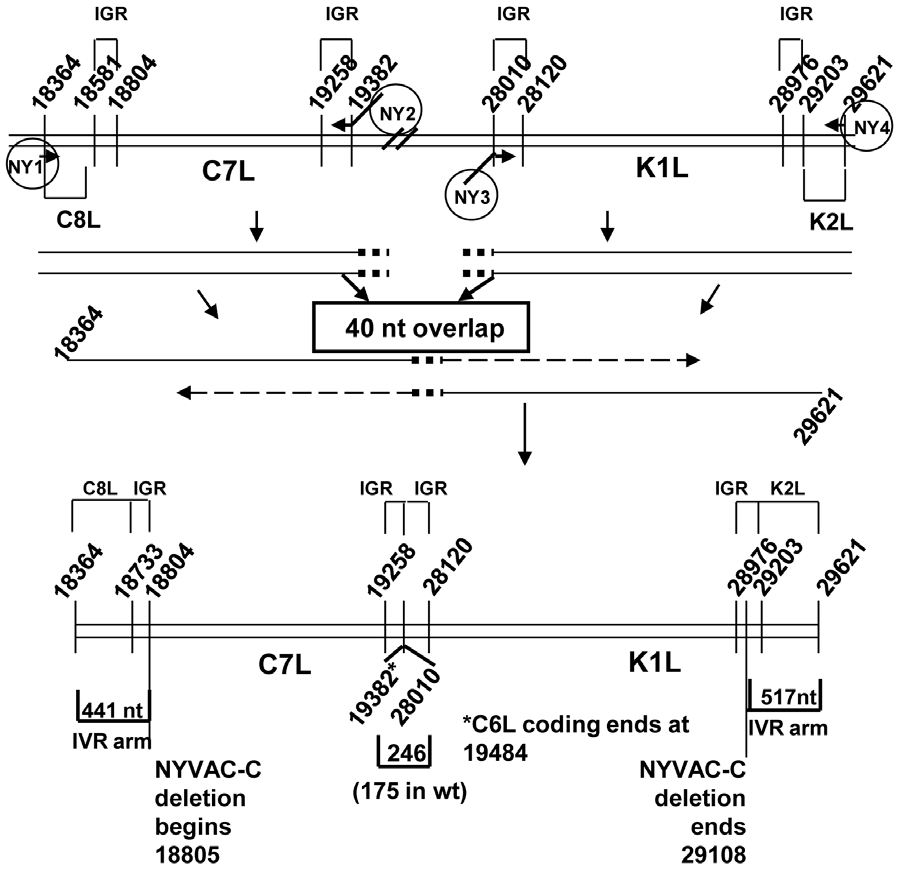
Scheme of construction of NYVAC-C-KC-ΔB19R. To insert *C7L* and *K1L* back into the NYVAC genome, each gene plus a corresponding portion of the flanking regions was amplified by PCR. The two fragments were combined into one fragment using PCR. The entire cassette, containing *C7L* and *K1L* plus flanking regions homologous to the adjacent genes of the NYVAC genome (*C8L* and *K2L*), was inserted into NYVAC-C by *in vivo* recombination. To create NYVAC-C-KC-ΔB19R, the KC fragment was inserted into NYVAC-C-ΔB19R by the same method. IGR: Intergenic region.

Because NYVAC has limited replication capacity in cultured human cells [14], our next goal was to produce a modified NYVAC vector able to complete the replication cycle in cultured human cells, but still maintaining an attenuated phenotype. *K1L* and *C7L* are two of the genes that were deleted during the construction of NYVAC, and these are both host range genes [23]. To restore replication in human cells, we inserted the *K1L* and *C7L* genes back into the NYVAC genome. Figure 2 shows a schematic diagram of the genome sequences assembled by PCR and inserted into the genome through IVR to create NYVAC-C-KC, NYVAC-C-KC-ΔB19R and NYVAC-C-KC-ΔB8RΔB19R.

### Replication in human cells

**Figure 3:**
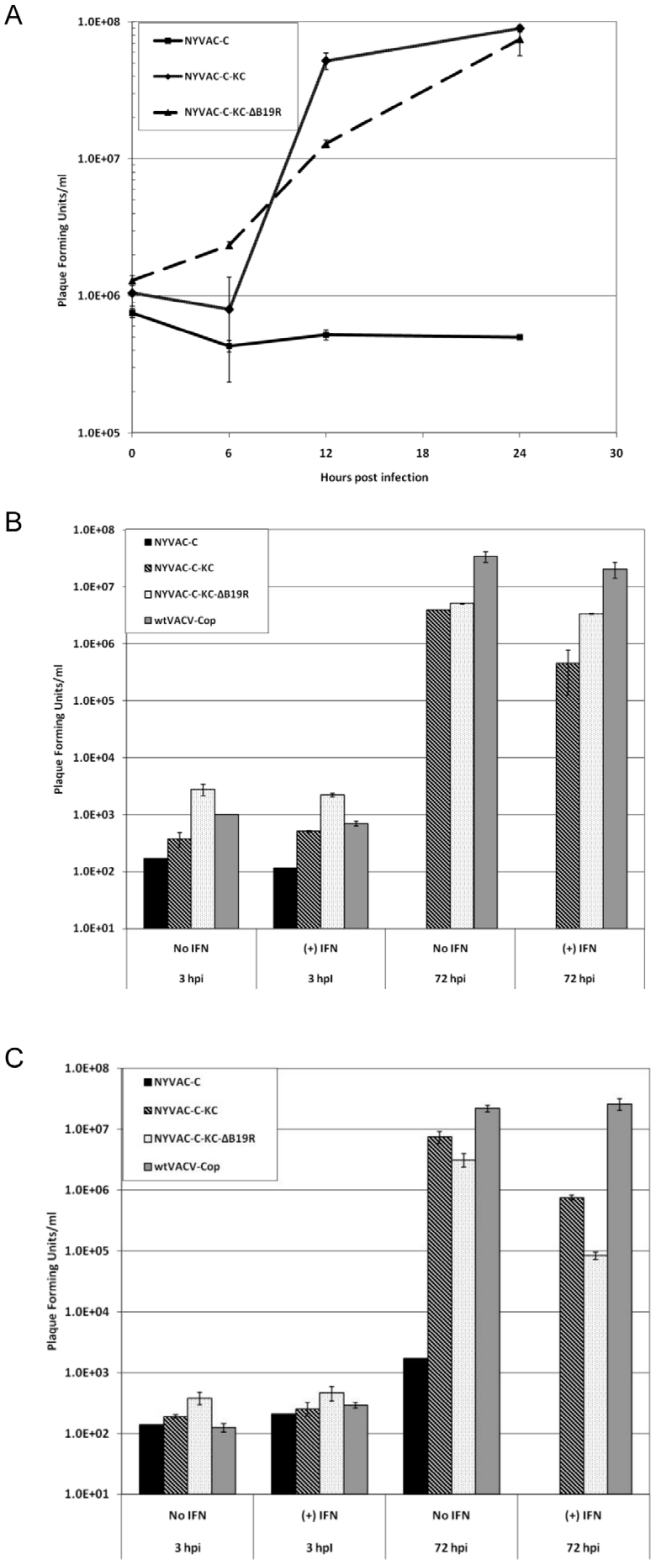
Replication capacity of NYVAC-C-KC and NYVAC-C-KC-ΔB19R in different human cells. **A) Single-step growth curve in HeLa cells.** Cells were infected with the indicated viruses at an MOI of 5 and harvested at 0, 6, 12, and 24 hours post-infection. Error bars are based on two separate infections. **B) Multi-step growth curves in human keratinocytes.** Cells were infected with the indicated viruses at an MOI of 0.01 and harvested at 3 or 72 hours post-infection, plus or minus 1000 Units/ml IFN. Error bars are based on two separate infections. **C) Multi-step growth curves in human dermal fibroblasts.** Cells were infected with the indicated viruses at an MOI of 0.01 and harvested at 3 or 72 hours post-infection, plus or minus 1000 Units/ml IFN. Error bars are based on two separate infections.

To verify that the presence of the two genes, *K1L* and *C7L*, in the modified virus does result in improved ability of the virus to replicate in human cells leading to higher levels of antigen expression, we examined first the replication capacity of NYVAC-C-KC compared to that of NYVAC-C, in a single step growth curve in HeLa cells (Fig. 3A). NYVAC-C-KC and NYVAC-C-KC-ΔB19R replicated under these experimental conditions in HeLa cells, but NYVAC-C did not. To be certain that the addition of the *K1L* and *C7L* genes would restore replication in human cell types that are physiologically relevant to poxvirus infection, we analyzed multi-step growth curves in human keratinocytes and human dermal fibroblasts and compared the NYVAC-C-KC replication in these primary cells (Fig. 3B and 3C) to that observed in HeLa cells (Fig. 3A). Though NYVAC-C did not replicate in any of the tested cells, NYVAC-C-KC and NYVAC-C-KC-ΔB19R replicated to titers comparable to that of the wtVACV-Cop in human keratinocytes and dermal fibroblasts (Fig. 3B and 3C). While wtVACV-Cop was fully resistant to treatment with IFN, NYVAC-C-KC and NYVAC-C-KC-ΔB19R showed increased sensitivity to IFN, especially in dermal fibroblasts. Thus, the addition of the *K1L* and *C7L* genes to NYVAC did restore replication competence in human cells.

### Transgene expression

**Figure 4:**
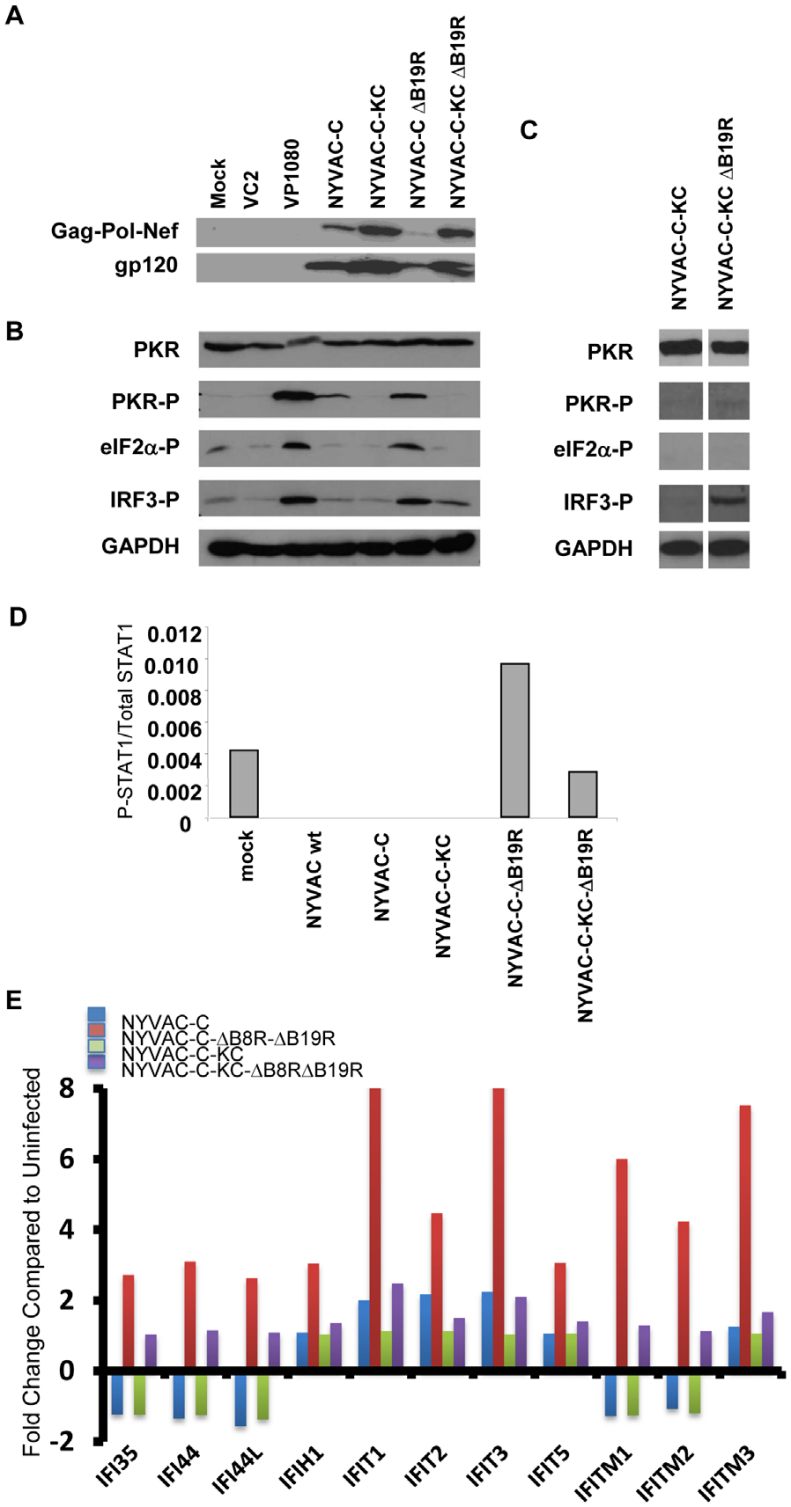
Signal transduction. **A**) Cells were infected at an MOI of 5; lysates were prepared at 6 hours post-infection and proteins were identified by a standard Western blot probed with anti-gp120 or anti-gag. **B**) HeLa cells were infected with the indicated viruses and harvested at 6 hours post-infection. Lysates were prepared and analyzed by Western blot to detect phosphorylation of shown signal transduction pathway components. **C**) Human keratinocytes were infected with the indicated viruses and harvested 6 hours post-infection. Lysates were prepared and analyzed by Western blot to detect phosphorylation of shown signal transduction pathway components. **D) Comparative analysis of P-STAT1 protein levels of non-replication vs. replication competent** ***B19R*** **deletion mutants.** Phospho-STAT1 levels present in extracts of cells infected with different recombinant viruses were measured by Cytometric Bead Array (CBA) using an LSR II flow cytometer. The concentrations of total and phospho STAT1 were measured simultaneously using the Cell Signaling Master Buffer Kit (BD Biosciences Pharmingen). For this CBA analysis, PBMCs were mock-infected or infected at an MOI of 5 with NYVAC wt, NYVAC-C, NYVAC-C-KC, NYVAC-C-ΔB19R or NYVAC-C-KC-ΔB19R. At 4 hours post-infection, cells were harvested and processed for CBA analysis as described in Materials and Methods and according to manufacturer's recommendations. Results are given as the ratio between phospho and total STAT1. **E) Microarray.** RNA was extracted from human pDCs infected with the indicated viruses. Data from a subset of the IFN-induced genes is shown. Fold increase (decrease) is normalized to results for mock-infected cells.

Insertion of *K1L* and *C7L* into NYVAC-C and deletion of *B19R* also had a dramatic effect on transgene expression. The *K1L* and *C7L* genes have been shown to decrease activation of PKR and inhibit the subsequent phosphorylation and inactivation of eIF2α [24], [25], [26]. This can be seen in Figure 4B. NYVAC-C infection induced an intermediate amount of PKR phosphorylation compared to infection with wtVACV-Cop (VC-2) on the one hand and virus deleted for the PKR inhibitor, *E3L* (vP1080), on the other hand. Insertion of *K1L* and *C7L* into NYVAC-C inhibited activation of PKR to background levels seen in VC2-infected cells. Inhibition of PKR activation in NYVAC-C-KC-infected cells was accompanied by an increase of expression of gag-pol-nef protein and gp120 in infected human cells, compared to cells infected with NYVAC-C (Fig. 4A). Deletion of *B19R* from NYVAC-C increased phosphorylation of PKR and *eIF2*α, nearly to the levels of virus deleted for *E3L*. This increase in both activation of PKR and phosphorylation of eIF2α was accompanied by a dramatic decrease in transgene expression. Insertion of *K1L* and *C7L* into NYVAC-C-ΔB19R reversed this trend, leading to a restoration of transgene protein synthesis to the level seen in cells infected with NYVAC-C-KC.

### Induction of Host Responses

Since B19 blocks the effects of type I IFN, we wanted to determine if deletion of *B19R* would have an effect on signaling downstream of IFN binding to its receptor on cells. The STAT-1 pathway, which is downstream of IFN induction, was not activated by infection with any virus containing *B19R* (wtNYVAC, NYVAC-C or NYVAC-C-KC, Fig. 4D). In fact, infection with any of these viruses led to decreased STAT-1 phosphorylation compared to mock-infected cells. However, deletion of *B19R* led to phosphorylation of STAT-1, even in the virus with *K1L* and *C7L* restored, compared to infection with the viruses containing *B19R*.

Several components of the virus sensing system are inducible by IFN (e.g., RIG-I and MDA-5). Since deletion of B19R can lead to IFN-induced signaling, we wanted to determine if these viruses affected phosphorylation of IRF-3. IRF-3 is a key transcription factor, which when phosphorylated and activated can lead to induction of type I IFN, and IFN is an important mediator between the innate and adaptive immune systems. Figure 4B shows the results of Western blot analysis of lysates from HeLa cells infected with either VC2 (wtVACV-Cop), vP1080 (CopΔE3L), NYVAC-C, NYVAC-C-KC, NYVAC-C-ΔB19R, or NYVAC-C-KC-ΔB19R to detect phosphorylation of IRF-3. wtVACV-Cop did not lead to phosphorylation of IRF-3, consistent with previously published results [27]. vP1080 (Copenhagen deleted of the *E3L* gene) was a strong activator of IRF-3 phosphorylation because the E3L protein is not present to mask the dsRNA [27]. Neither NYVAC-C nor NYVAC-C-KC induced detectable phosphorylation of IRF-3. However, deletion of *B19R* from either of these viruses resulted in phosphorylation of IRF-3. Results in human keratinocytes (Fig. 4C) were similar to those in HeLa cells, although we did observe donor-to-donor variation in the activation patterns (data not shown).

The signal transduction results were extended by microarray analysis of infected cells, as shown in Figure 4E. The detailed microarray data from human dendritic cells (DCs) infected with the NYVAC vectors has been recently described [8]. Shown here is representative data from DCs infected with either NYVAC-C or NYVAC-C-KC, or either of those viruses deleted also of both *B8R* and *B19R*. The results from infection with this double-deletion mutant were indistinguishable from those of the single deletion of *B19R* only (data not shown). Infection with either NYVAC-C or NYVAC-C-KC induced very little change to any of the shown IFN-inducible genes, consistent with the IRF-3 phosphorylation and STAT-phosphorylation results shown in Figure 4B and 4D. The deletion of *B8R* and *B19R* from NYVAC-C results in a strong inflammatory response, as evidenced by the transcriptional up-regulation of all the genes shown on the graph, again consistent with the results seen in Figure 4B and 4D. With the insertion of *K1L* and *C7L*, this response was tempered; infection with NYVAC-C-KC-ΔB8RΔB19R induced increased transcriptional levels of seven of the genes shown, relative to NYVAC-C-KC (IFI35, IFI44, IFI44L, IFIT1, IFIT3, IFITM1, and IFITM2). The lower level of induction of these genes by NYVAC-C-KC-ΔB8RΔB19R compared to that of NYVAC-C-ΔB8RΔB19R is consistent with signal transduction data, since NYVAC-C-KC-ΔB8RΔB19R induced less IRF-3 and STAT-1 phosphorylation than did NYVAC-C-ΔB8RΔB19R (data not shown).

**Figure 5:**
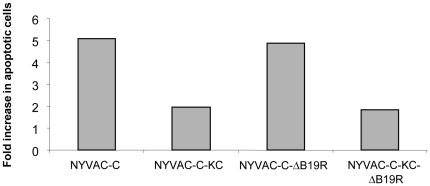
Comparative analysis of the apoptosis induction of non-replication versus replication-competent *B19R* deletion mutants. The different stages of cell cycle and the percentage of cells with subG_0_ DNA content were analyzed by propidium iodide (PI) staining and FACS analysis. HeLa cells were mock-infected or infected at an MOI of 5 with NYVAC-C, NYVAC-C-KC, NYVAC-C-ΔB19R or NYVAC-C-KC-ΔB19R. At 24 hours post-infection cells were harvested and processed as described in Materials and Methods. The percentage of cells with hypodiploid DNA content was determined using an LSR II flow cytometer (Becton Dickinson). The results are expressed as fold increase in apoptotic cells with respect to uninfected cells.

Since activation of PKR has previously been shown to lead to induction of apoptosis, we assayed for the ability of these modified viruses to induce apoptosis. Consistent with the activation of PKR in HeLa cells (Fig. 4B), apoptosis in HeLa cells infected with NYVAC-C or NYVAC-C-ΔB19R was increased when normalized to uninfected cells, while the level of apoptosis was not increased in cells infected with the viruses containing the *K1L* and *C7L* genes (Fig. 5).

### Safety profile

**Figure 6:**
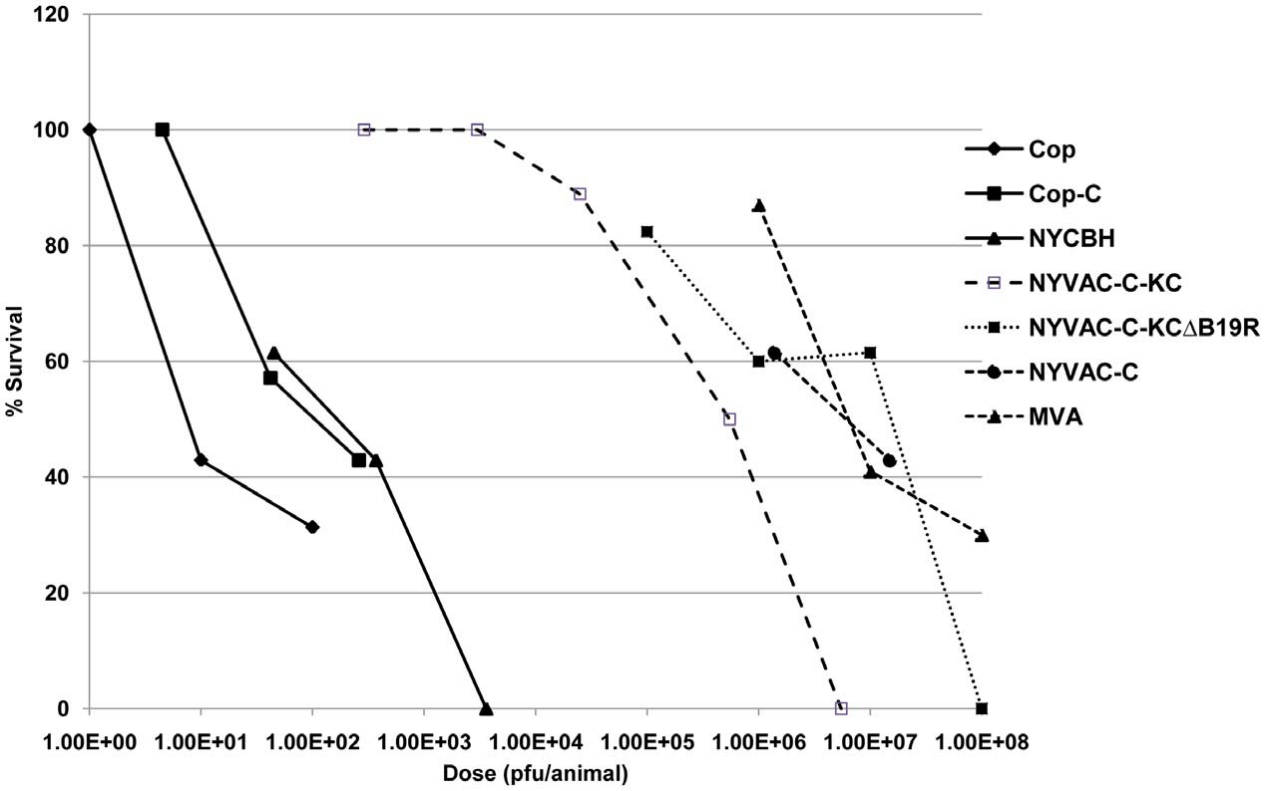
Pathogenesis study. Newborn CD1 mice (minimum of 10 per group) were inoculated IC with the indicated viruses at the indicated doses of plaque forming units (pfu). Mice were monitored twice daily for 14 days for signs of morbidity/mortality.

Though our goal was to improve the parental NYVAC vaccine vector, it was equally important to maintain sufficient safety levels with the pox vector. To compare the pathogenicity of NYVAC-C-KC constructs to existing vaccines, we utilized a newborn mouse model, the most sensitive mouse model available for detecting pox virus pathogenesis [11]. Newborn mice were inoculated intracranially (IC) with the indicated viruses. As can be seen in Figure 6, wtVACV-Cop was highly pathogenic in this model, with an LD_50_ of only 10 pfu. VACV-CopC, with the loss of the TK gene, is further attenuated by about one log, and has an LD_50_ comparable to that of the New York City Board of Health (NYCBH) [28] strain of VACV, the strain of VACV currently used for vaccination against smallpox. NYVAC-C-KC was attenuated by approximately four logs compared to NYCBH. Deletion of *B19R* from NYVAC-C-KC further attenuated the virus by approximately one log, approaching the attenuation of MVA and NYVAC-C. Thus, the safety profile of NYVAC-C-KC-ΔB19R, despite this construct being nearly fully replication-competent in primary human skin cells in culture, is in the same general range as the highly attenuated, replication-restricted vaccinia virus strains, MVA and NYVAC-C.

Since most of the complications due to use of vaccinia virus require spread from the site of inoculation, we followed two approaches to characterize spread of the virus in infected animals: one determining virus titers in different organs and the second, evaluating viral expression by the levels of the marker luciferase in NYVAC recombinants.

**Figure 7:**
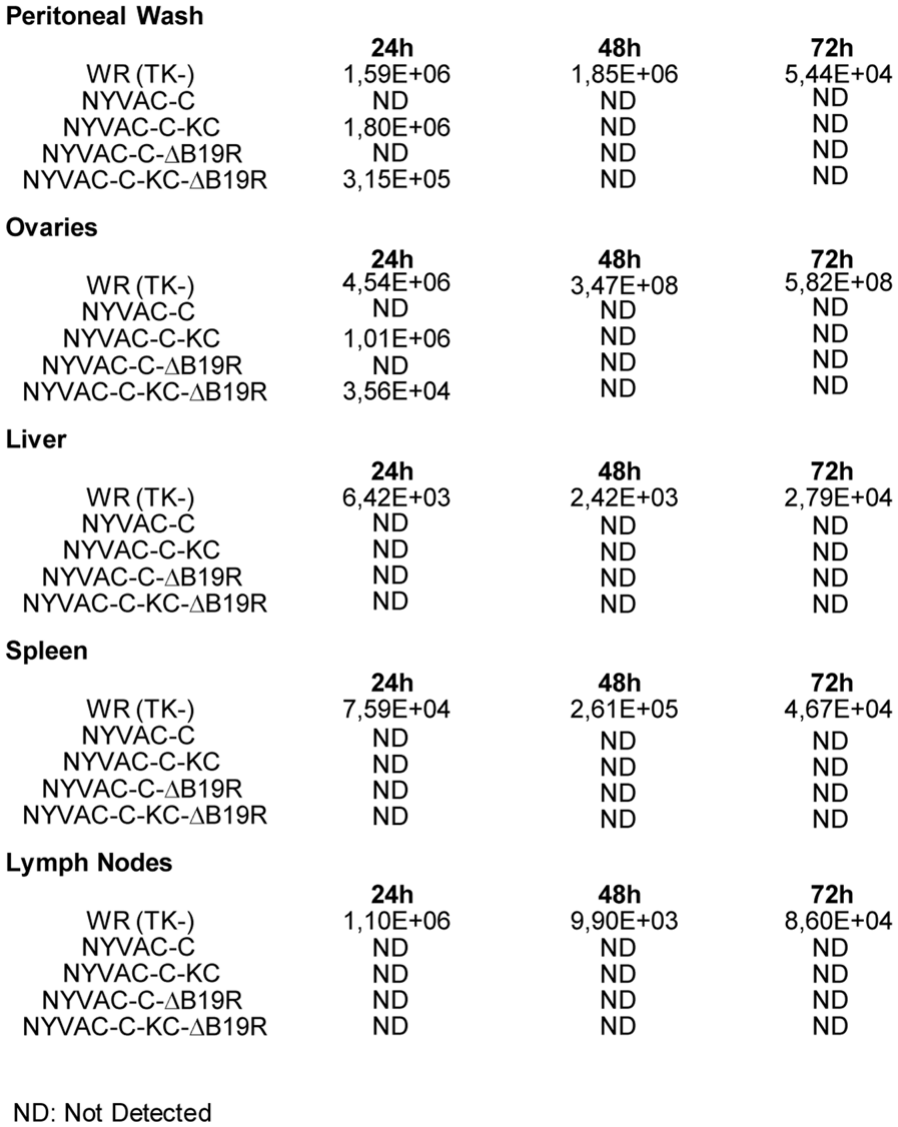
Comparative analysis of the biodistribution of non-replication versus replication-competent *B19R* deletion mutants. Five groups of mice (n = 6) received an IP inoculation of 2×10^7^ PFU/mouse of WR (TK^−^), NYVAC-C, NYVAC-C-KC, NYVAC-C-ΔB19R or NYVAC-C-KC-ΔB19R. At different times post-inoculation (24, 48 and 72 hours) animals were sacrificed and different mouse tissues (peritoneal cells, ovaries, livers, spleens and draining lymph nodes) were harvested and processed for titration as described in Materials and Methods. The production of infectious virus was determined by plaque assay in BSC-40 cells. The virus titer was expressed as Plaque Forming Units (pfu) per gram of protein.

To measure titers in different organs, mice were inoculated intraperitoneally (IP) with 2×10^7^ pfu/mouse of virus and at different days post infection the animals were sacrificed and the presence of virus in the peritoneal wash, ovaries, liver, spleen, and lymph nodes was measured. As shown in Figure 7, the WR strain of VACV was detectable at high titers in all tissue samples (peritoneum, ovaries, liver spleen and lymph nodes), even up to 72 hours post-infection. Infectious NYVAC-C or NYVAC-C-ΔB19R was not detectable in any tissue at any time. Both NYVAC-C-KC and NYVAC-C-KC-ΔB19R were transiently detectable in the peritoneum and ovaries, but resolved by 48 hours post-infection. Levels of NYVAC-C-KC were similar to wtVACV-WR in the peritoneal cavity, but 4- to 5-fold lower than wtVACV-WR in ovaries. NYVAC-C-KC-ΔB19R was detectable in 5-fold and 100-fold lower amounts in the peritoneal cavity and ovaries, respectively, compared to wtVACV-WR.

**Figure 8:**
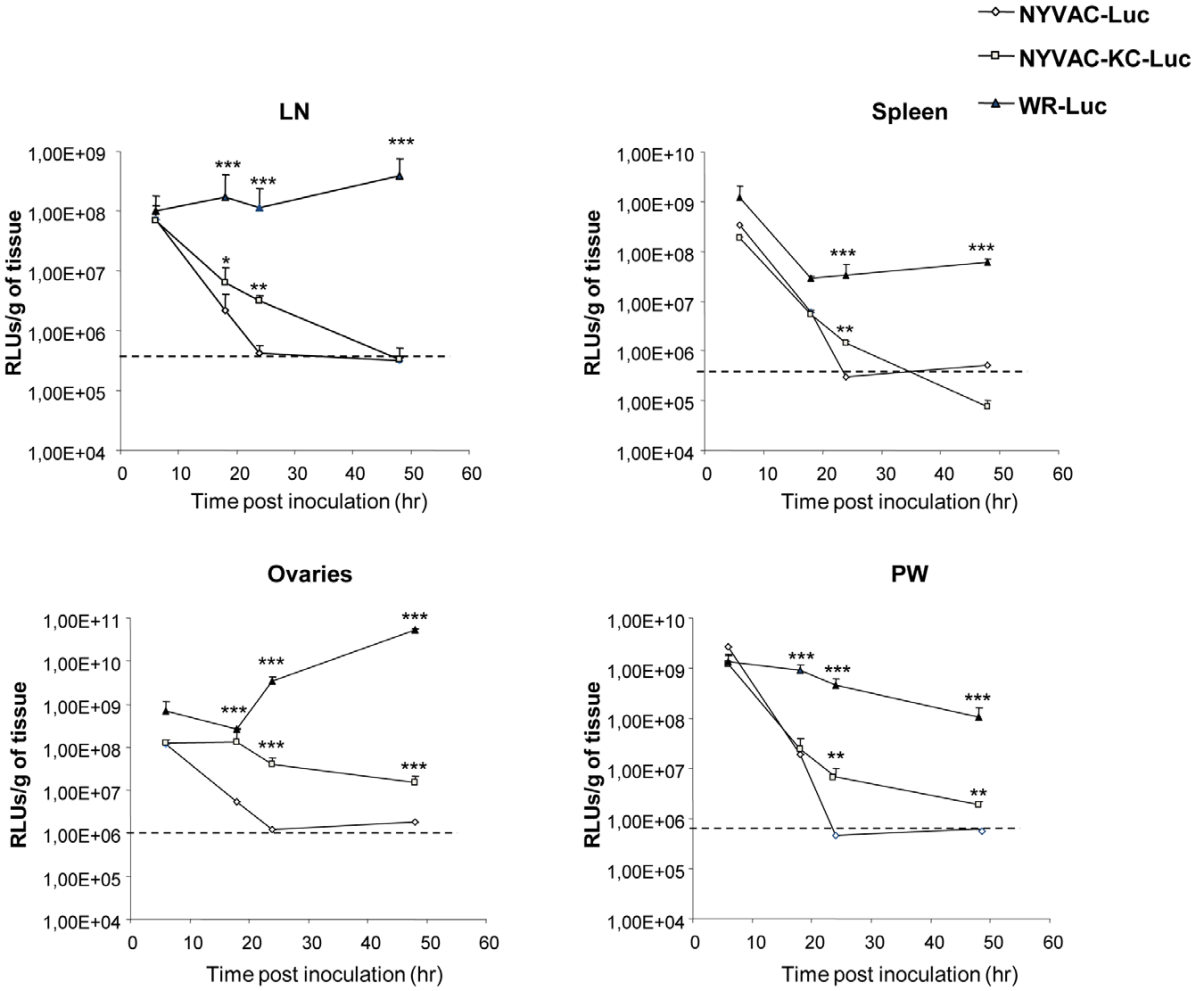
Comparative levels of luciferase in mice infected with parental NYVAC versus NYVAC-C-KC. Mice were infected IP with 2×10^7^ plaque forming units/mouse of viruses (WR-luc, NYVAC-luc and NYVAC-KC-luc) expressing the luciferase marker and at different times several tissues were harvested, lysed and luciferase activity was determined as previously described [12], [13]. Values are represented as reference luciferase units per gram of tissue.

These results were confirmed with recombinant viruses expressing luciferase. Groups of mice were infected IP with 2×10^7^ pfu/mouse of WR-luc, NYVAC-luc or NYVAC-KC-luc and at different time points, levels of luciferase were evaluated in mouse tissues. As shown in Figure 8, luciferase expression remained elevated in animals infected with the fully replication-competent WR-luc, while the levels were reduced in NYVAC-infected animals. Luciferase expression was intermediate for animals infected with NYVAC-KC-luc.

## Discussion

An important consideration in the HIV/AIDS vaccine field is the nature of the vectors needed for protective efficacy. While numerous different combinations of vectors are being considered as vaccine regimens, one combination has been shown to be partially effective. This has been shown in the phase III Thai clinical study (RV144) with the vaccine combination of a canarypox virus (ALVAC) and the monomeric protein gp120, giving 31% protection against HIV infection [7]. The modest efficacy of the RV144 trial suggests that improvement of the vaccination protocol is possible. Since ALVAC is a replication-restricted virus in human cells and the other candidate VACV vectors, MVA and NYVAC, are also replication-restricted, it is possible that improvements of these vectors might be achieved through enhancement of their replication capacity. In this investigation we have achieved this goal by the re-insertion of the host restriction genes *K1L* and *C7L* into the NYVAC genome. Re-insertion of these two genes restored replication capacity in human cultured cells, including physiologically relevant primary keratinocytes and primary dermal fibroblasts. A concern of replication-competence in human cultured cells has been tempered by the demonstration that the replication-competent viruses described in this manuscript are highly attenuated compared to other replication-competent strains. This has been demonstrated through intracranial inoculation of the vectors in newborn mice. Clearly the vector NYVAC-C-KC is highly attenuated compared to the Copenhagen and NYCBH strains (Fig. 6). The attenuation was further improved by deleting the type I IFN inhibitor B19. The likely reason that increased attenuation is associated with deletion of *B19R* is that type I IFN induced during virus infection is no longer blocked by the virus inhibitor B19 and, hence, pro-inflammatory pathways are more readily activated (Fig. 4).

The consequences of the incorporation of the host genes *K1L*-*C7L* into NYVAC-C include the enhanced expression of foreign antigens, such as the HIV-1 protein gp120 and the fusion protein gag-pol-nef in cultured cells (Fig. 4A). Viral protein expression in vaccinia virus-infected cells is often regulated by activation of the cellular protein, PKR, which when activated, effectively inhibits translation of viral proteins. Poxviruses use several mechanisms to block activation of PKR, including the dsRNA binding protein E3L [27], [29]. However, work done by others has shown that *K1L* is also necessary to prevent activation of PKR in vaccinia virus-infected cells [24], [30], and that even expression of early viral proteins is down-regulated in the absence of *K1L*. Therefore, antigen expression by the viral vector is enhanced by the presence of *K1L*. We also detected increased luciferase expression in animals infected with NYVAC-KC-luc. In this case, the increased level of antigen expression may result from a combination of restored replication-competence which likely leads to an increase in the number of cells infected in the animal, and to a *de novo* increase in translation in infected cells in the animal. In the absence of a mechanism to separate replication-competence from increased protein expression in the individual cell, it is difficult to ascertain the contribution of each.

It is curious that deletion of *B19R* has such a dramatic effect on activation of PKR and phosphorylation of IRF-3. Our hypothesis is that B19 normally blocks the priming effect resulting from small amounts of IFN secreted from the infected host cell. In the absence of B19, this priming effect is allowed to take place, inducing pathogen-associated molecular pattern (PAMP) sensors, such as RIG-I and PKR, and initiating signal transduction pathways that may enhance the adaptive immune response against the antigen. The highly-attenuated phenotype of the NYVAC-C-KC-ΔB19R virus, along with higher antigen expression levels than observed with its parental virus NYVAC-C, and its ability to induce pro-inflammatory signaling and pro-inflammatory gene expression, suggest that this virus provides a potential improved vaccine vector for human application.

Previous studies on the immunological characteristics of NYVAC-C have been documented in different models: mouse [15], [16], macaque [17] and human clinical trials [18], [19]. The results obtained thus far indicate that NYVAC-C is immunogenic but needs further improvements. In fact, prime/boost combination with DNA vectors is needed to expand the breadth and strength of the immune responses to HIV antigens. In the phase I clinical studies, two doses of NYVAC-C given intramuscularly resulted in positive responses to HIV antigens in fewer than 40% vaccinees [18], [19]. It was only after priming with DNA vectors that responses rose to about 90% of vaccinees [18]. It is likely that the limited expression of HIV antigens and the replication-restriction of NYVAC-C in human cells played an important role in the observed limitations in the immune responses. The use of NYVAC-K-KC or NYVAC-C-KC-ΔB19R might represent an important advance in improving NYVAC-based vaccine vectors. This has been further supported by additional studies with these vectors showing activation of pathways involved in antigen processing and presentation, enhanced cross-presentation to HIV-specific CD8 T cells, and proliferation of specific memory CD8 T cells *in vitro* [8].
